# Genetic Diversity of Bovine Hemoprotozoa in South Korea

**DOI:** 10.3390/pathogens9090768

**Published:** 2020-09-20

**Authors:** Dongmi Kwak, Min-Goo Seo

**Affiliations:** 1 College of Veterinary Medicine, Kyungpook National University, Daegu 41566, Korea; dmkwak@knu.ac.kr; 2 Cardiovascular Research Institute, Kyungpook National University, Daegu 41944, Korea; 3 Veterinary Drugs and Biologics Division, Animal and Plant Quarantine Agency, Gyeongbuk 39660, Korea

**Keywords:** cattle, piroplasm, phylogeny, *Theileria*

## Abstract

Tick-borne pathogens cause economically significant diseases in cattle. *Theileria* spp. are parasitic protozoa and the causative agent of bovine theileriosis. Here we report the distribution and risk factors of bovine *Theileria* using blood samples taken between 2018 and 2019. Of 737 tested cattle, nine animals (1.2%) were positive for *Theileria orientalis* infection by 18S rRNA gene amplification. Further analysis of the infected samples using the *T. orientalis* major piroplasm surface protein (MPSP) gene revealed five different genotypes circulating in the population: Types 1, 2, 3, 7, and N3. To the best of our knowledge, this is the first research to describe the existence of the *T. orientalis* MPSP genotype N3 in South Korea. Although the prevalence of bovine *T. orientalis* was low, our study offers data on the geographical distribution and prevalence of bovine *Theileria* spp. in South Korea. Further studies are warranted to determine the correlation of clinical symptoms with parasite MPSP genotypes. Our data provide epidemiological information to help control bovine theileriosis in South Korea.

## 1. Introduction

Tick-borne pathogens (TBPs) cause significant morbidity and mortality in mammals, representing a major public health threat and damaging livestock production [1]. The piroplasms *Babesia* and *Theileria* are widely distributed intracellular parasitic protozoans that infect erythrocytes, or erythrocytes and lymphocytes, respectively, and are economically significant TBPs of domestic and wild animals [2].

*Theileria* spp. (Apicomplexa: Piroplasmida: Theileriidae) are transmitted by ticks and cause bovine theileriosis, an economically important disease in cattle [3]. Infected animals display a range of symptoms, including chronic anemia, fever, reduced milk production, weight loss, anorexia, and jaundice [4]. Several species of *Theileria* infect ruminants, with a wide variation in pathogenicity. *Theileria parva* and *T. annulata* are known to cause East Coast fever and tropical theileriosis, respectively. Both species are highly pathogenic in cattle. A third species, *T. orientalis*, was previously believed to induce only mild or asymptomatic disease [5]; however, it has recently emerged as a virulent parasite that can cause clinical theileriosis and has resulted in notable economic impact to the Asia-Pacific cattle industry [6]. These species, including *T. buffeli, T. orientalis*, and *T. sergenti,* are considerably similar, and the discrete taxonomy of this group is debatable. On the basis of molecular approaches, the three species of parasites are currently categorized as one species, namely *T. orientalis*; however, a study has proposed that it should be called the *T. orientalis* complex [4]. *T. orientalis* belongs to a comparatively benign group of *Theileria* with universal existence. It causes little pathogenicity in bovine compared with *T. parva* and *T. annulata;* however, it has caused considerable economic loss in China [7], South Korea [8,9,10,11], Japan [12], Australia [6], and Vietnam [13] due to decreased production of meat, milk, and other by-products.

The active areas and prevalence of ticks are important factors in the epidemiology of *Theileria* species in cattle [7]. In South Korea, *Haemaphysalis longicornis*, which is the most prevalent tick species, is a primary vector of *T. orientalis* [14,15]. *T. orientalis* has been detected in *H. longicornis* infesting cattle in South Korea [15]. Although *T. orientalis* causes low virulence in healthy cattle, in endemic areas, older cattle with subclinical theileriosis may remain long-term carriers of piroplasm and may act as a reservoir of infection for tick and other animals [5,8]. Here, we investigated the distribution and risk factors of bovine *T. orientalis* in South Korea and collected epidemiological information needed to control piroplasm infections in South Korea.

## 2. Results

### 2.1. PCR and Molecular Identification

We used the 18S rRNA sequences of *T. orientalis* to detect infections in cattle (9/737, 1.2%; 95% confidence interval (CI): 0.4–2.0). Additional genetic analysis of the positive samples revealed that the cattle were also positive for the *T. orientalis* major piroplasm surface protein (MPSP) gene (9/737, 1.2%; 95% CI: 0.4–2.0) (Table 1). The prevalence of cattle positive for *T. orientalis* varied for each group (Table 1). Female cattle (*p* = 0.0293) had a greater possibility to be positive for *T. orientalis* than male cattle. In addition, cattle over 4 years old (*p* = 0.0176) had a greater possibility to be positive for *T. orientalis* than other age groups. Moreover, we detected differences in breed and region susceptibilities. Only the Holstein breed (*p* = 0.0079) tested positive for *T. orientalis*, with no infections detected in the native Korean brown cattle, Hanwoo breed. On the other hand, the Gyeongnam Province cattle (*p* = 0.0451) were positive for *T. orientalis*, with no infections detected in cattle from the Gyeongbuk Province. Furthermore, only samples taken in summer (*p* = 0.0166) were positive for *T. orientalis* compared with those in other seasons. *Babesia* spp. was not detected in this study.

### 2.2. Molecular Analyses

**Figure 1:**
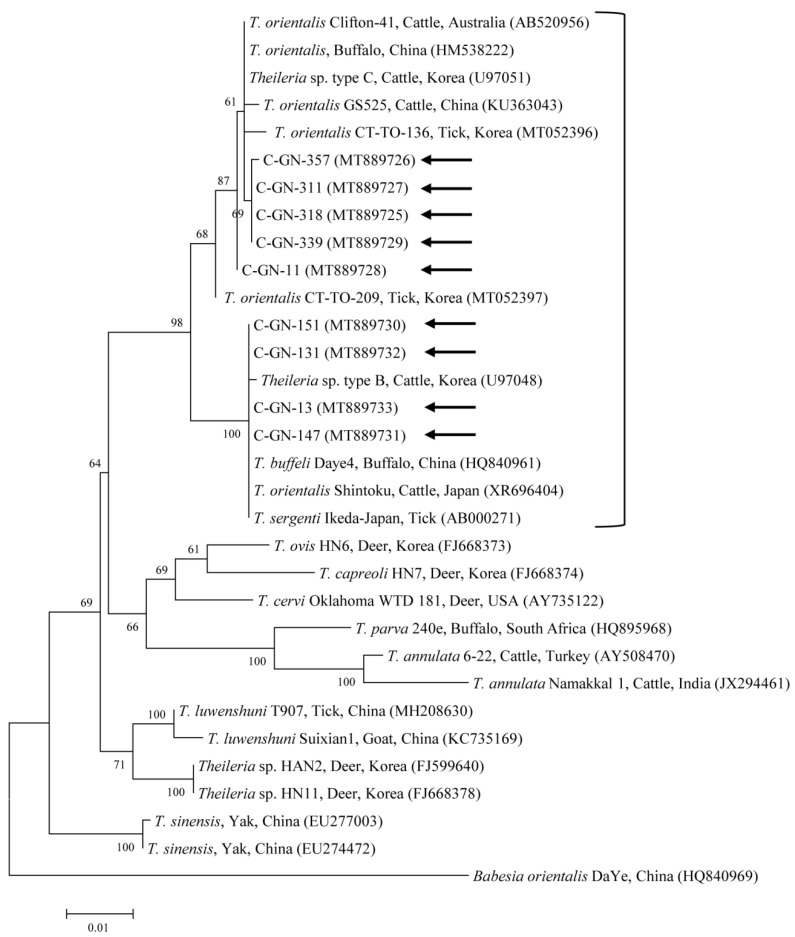
Phylogeny of *Theileria* spp. on the basis of 18S rRNA nucleotide sequences created using the maximum-likelihood method. Black arrows denote the sequences reported in the current study. The GenBank accession numbers of the other sequences are shown in parentheses. *Babesia orientalis* is used as the outgroup. Bootstrap support levels (1000 replicates) are shown by branch numbers and the number of substitutions for each nucleotide is indicated by the scale bar.

**Figure 2:**
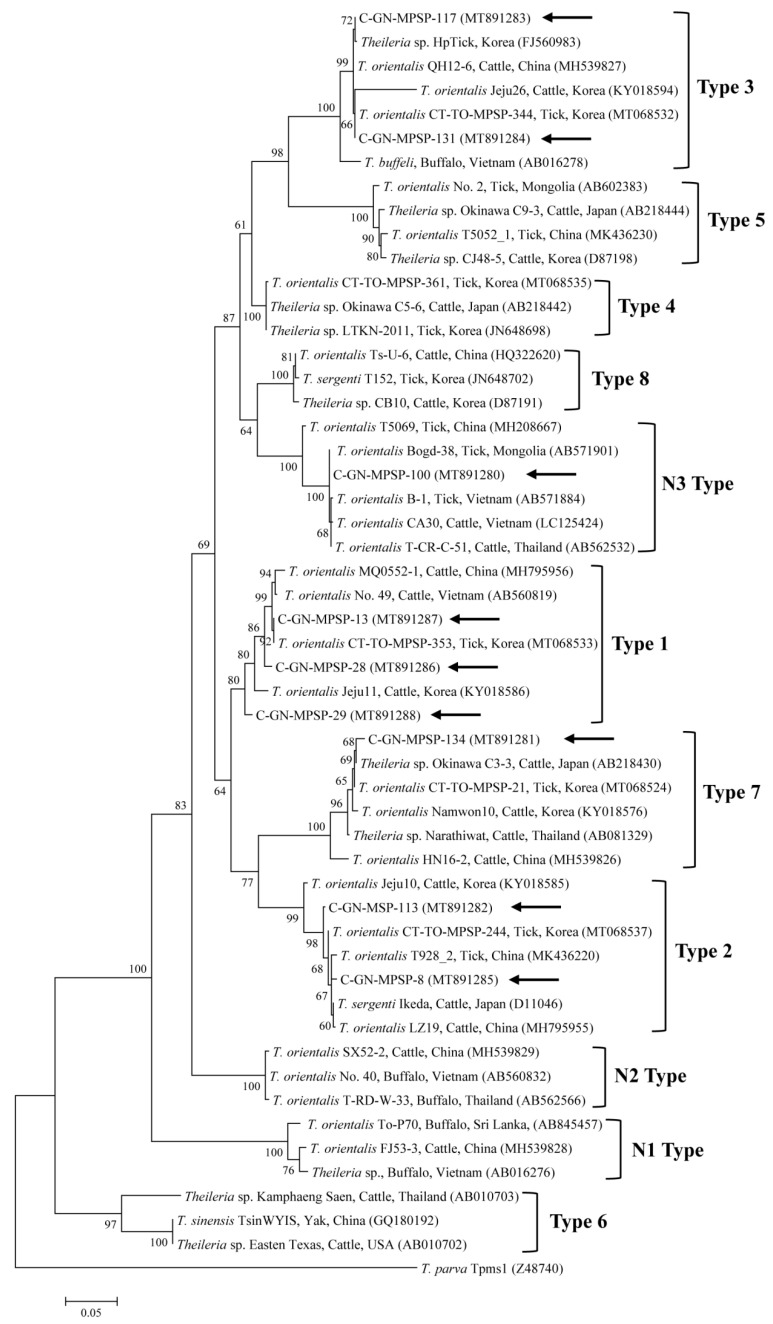
Phylogeny of *Theileria* spp. on the basis of MPSP gene sequences constructed using the maximum-likelihood method. Black arrows denote the sequences described in the current study. The GenBank accession numbers of all other sequences are given in parentheses. *Theileria parva* is used as the outgroup. Bootstrap support levels (1000 replicates) are shown by branch numbers and the number of substitutions for each nucleotide is indicated by the scale bar.

Phylogenetic analysis revealed that the *T. orientalis* 18S rRNA (Figure 1) and MPSP (Figure 2) nucleotide sequences obtained in this study were clustered with the previously deposited *T. orientalis* sequences in GenBank, as shown in Figure 1 and Figure 2.

The nine *T. orientalis* 18S rRNA sequences detected in this study had 97.7–99.9% identity with each other. They also shared 97.5–99.9% identity with those of previously reported *T. orientalis* 18S rRNA sequences in GenBank. The *T. orientalis* MPSP nucleotide sequences were categorized into five genotypes: Types 1, 2, 3, 7, and N3. Among the nine sequences, three, two, two, one, and one isolates were assigned to types 1, 2, 3, 7, and N3, respectively. The three, two, and two MPSP sequences of types 1, 2, and 3 detected in this study had 97.5–98.4%, 98.8%, and 99.6% identity with each other, respectively. In addition, they shared 98.5–99.8%, 99.0–99.5%, and 98.1–100% identity with the *T. orientalis* MPSP nucleotide sequences previously reported in GenBank, respectively. The single sequences of types 7 and N3 that we found each shared 98.2–98.9% and 98.6–99.3% identity with those *T. orientalis* MPSP nucleotide sequences previously deposited in GenBank, respectively. The representative sequences reported in the current study were deposited to GenBank, with the following accession numbers: MT889725–MT889733 (*Theileria* spp. 18S rRNA) and MT891280–MT891288 (*Theileria* spp. MPSP).

## 3. Discussion

Theileriosis is one of the most significant hemoprotozoan diseases infecting domestic animals, most commonly cattle and sheep, in tropical and subtropical regions and results in significant economic loss [3].

Recently, *T. orientalis* was detected in samples from South Korea using the 18S rRNA gene in ticks (3.7%, 21/566 pools) infesting cattle in 2010–2011 [14], in *H. longicornis* ticks (5.0%, 29/576) from cattle in 2014–2018 [15], in cattle (21.7%, 96/443) in 2009 [11], and in cattle (23.2%, 69/298) in 2014–2015 [8]. Compared with other previous studies in South Korea, we detected a relatively low prevalence of *T. orientalis* in cattle (1.2%, 9/737). This may be due to differences in the cattle breeds, region, seasons, climate, and ticks.

The prevalence of *T. orientalis* was significantly higher in female cattle than in males. Males are mainly reared for beef production in South Korea; they are usually bred for less than three years. On the other hand, females are reared for reproduction and dairy production and have a longer lifetime and, subsequently, longer exposure period to ticks. In the present study, female dairy cattle had a higher *T. orientalis* prevalence than male dairy cattle. These results are similar to those of a previous study in South Korea, which reported that the prevalence of *T. orientalis* in female cattle (23.3%, 94/403) was higher than that in male cattle [11].

*T. orientalis* was only detected in dairy cattle. The reason for this could be due to differences in cattle management and breeds. Dairy cattle are typically raised in barns near grassland regions in South Korea; therefore, they are more susceptible to contacting ticks. Furthermore, only cattle from two farms were infected with *T. orientalis.* All the infected cattle were permitted to graze near grassland regions, exposing them to ticks. Similar results were reported previously in South Korea, where the prevalence of *T. orientalis* in dairy cattle (27.9%, 82/294) was higher than in other breeds [11]. In the current study, significant differences in the prevalence of bovine *T. orientalis* were observed with age. Older cattle had a higher *T. orientalis* prevalence compared with young cattle. This tendency in the age-involved prevalence was same as that in a previous cattle study [11].

*T. orientalis* infections were only detected in the cattle raised in the Gyeongnam Province. The Korean Peninsula is steadily shifting to a subtropical climate [16]. Moreover, the Gyeongnam Province is at a lower latitude in the mainland than in the Gyeongbuk Province. This biogeoclimatic difference could possibly explain the detected differences in the prevalence of TBPs and ticks. For instance, cattle were positive for *T. orientalis* (96.2%, 25/26) from Jeju Island, which is at a lower latitude in South Korea [9]. In addition, TBPs including *T. orientalis* were detected in several ticks in varying proportions in different areas, and TBP infections in ticks were more abundant in the southern region than other regions in South Korea, in both the nymph and adult stages [15]. The higher prevalence of *T. orientalis* infection at the lower latitude may also be due to environmental changes, such as humidity, rainfall, and temperature, due to climate change, resulting in increased tick activity and reproduction [9].

Most regions of South Korea are mountainous, and summer provides the ideal conditions for ticks. A previous study has suggested that the prevalence of *Theileria* infection is more related to the season, mainly to the emergence of ticks and their activity levels [17]. A notable increase in tick infestation in the mountainous regions during summer may induce increased *T. orientalis* infection rates, resulting in notable changes in red blood cell profiles after grazing [17]. This study also presented significantly high prevalence of *T. orientalis* in summer.

The MPSP gene is conserved among different geographic isolates and is expressed in the intraerythrocytic stage of *T. orientalis*. As a result, it is the most frequently used marker and has been used for phylogenetic and epidemiological analyses [4]. The *T. orientalis* MPSP gene sequence comprises at least 11 different MPSP genotypes (types 1–8 and N1–N3) in sheep, cattle, ticks, and water buffaloes [4]. Of the 11 MPSP genotypes, *ikeda* (type 1), *chitose* (type 2), *buffeli* (type 3), types 4–8, and N1–N3 have been reported globally [4]. Among them, type 6 has been detected in cattle and yaks and is identified as *T. sinensis* [18]. 

Recently, genotype studies in South Korea have detected *Theileria* MPSP genotypes 1, 2, 4, and 8 in ticks (2.7%, 15/556) from grazing cattle [19]; types 1–4 and 7 (5.0%, 29/576) in ticks from cattle [15]; types 1, 2, and 7 (17.7%, 12/68) in cattle [10]; and types 1–3 and 7 (41.3%, 57/138) in cattle [9]. In China, types 1–5, 7, N3, and novel N4 of the *Theileria* MPSP gene (4.5%, 67/1488) were detected in ticks [20] and types 1–5, 7, N1, and N2 of the *Theileria* MPSP gene (36.5%, 95/260) were detected in cattle [21]. In this study, types 1–3, 7, and N3 of the *Theileria* MPSP gene (1.2%, 9/737) were detected in cattle. To the best of our knowledge, this is the first study to describe the presence of *Theileria* MPSP genotype N3 in South Korea. However, among the nine MPSP genes isolated, only one sequence is genotype N3.

Of the five MPSP genotypes identified, type 1 (33.3%, 3/9) was the most commonly identified genotype in ticks. In previous studies in South Korea, type 1 in cattle [10], type 2 in cattle [9], types 1 and 2 in ticks infesting cattle [15], and types 2 and 4 in ticks infesting cattle [19] were also prominent. These results suggest that types 1 and 2 are distributed nationwide and maybe related to a potential risk of theileriosis in South Korea.

Among the 11 genotypes, to date, only MPSP types 1 (*chitose*) and 2 (*ikeda*) have been associated with the clinical form of oriental theileriosis. Of these, type 2 is more pathogenic in cattle, causing severe anemia, parasitemia, and death [4]. As the clinical presentation of cattle was beyond the scope of the present study, further studies are warranted to identify the correlation between clinical symptoms and pathogenic strains of *T. orientalis*.

In addition to genetic variability, some risk factors were also analyzed in the present study. However, further studies are warranted to assess the regional distributions and epidemiological importance of genetic divergence in *Theileria* isolates. This information will help in developing efficient control and prevention methods for oriental theileriosis. This is the first report of the MPSP genotype N3 in South Korea, increasing our understanding of the genetic diversity of *T. orientalis* in domestic livestock. Although the prevalence of bovine *T. orientalis* infection was low, the present study provides data on the geographical distribution and prevalence of bovine theilerioses in this region.

## 4. Materials and Methods

### 4.1. Ethics Statement

This research was performed between 2018 and 2019 and did not require approval from the Institutional Animal Care and Use Committee at Kyungpook National University, which regulates laboratory animals maintained in indoor facilities only. Blood samples were collected from cattle by practicing veterinarians at local clinics, during periodic medical checkups, after verbal consent from farmers.

### 4.2. Samples

In 2019, 3,645,190 cattle were reared on 100,175 farms in South Korea, including 750,686 (20.6%) cattle on 20,822 (20.8%) farms in Gyeongbuk Province and 362,464 (9.9%) cattle on 13,566 (13.5%) farms in Gyeongnam Province [22]. The sample size was decided statistically using a formula with an expected disease prevalence of 10%, a confidence level of 95%, and an accepted absolute error of 5% using a simple random sampling strategy [23].

**Figure 3:**
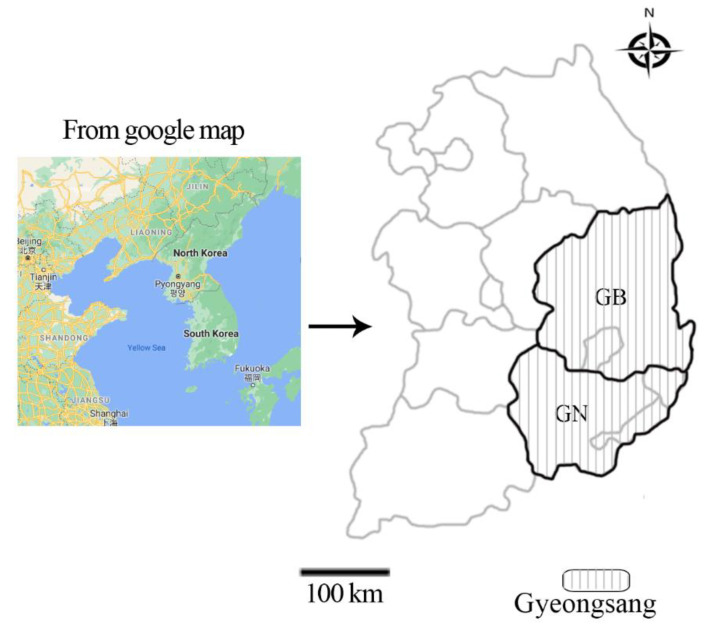
Map of South Korea showing the regions from which the cattle blood samples were collected for the detection of *Theileria* spp. from the Gyeongsang region (vertical lines), including Gyeongbuk (GB) and Gyeongnam (GN) Provinces.

In accordance with the formula, at least 138 samples were required. In this study, 737 cattle (Hanwoo, native Korean brown cattle; Holstein, dairy cattle) blood samples were collected from 41 cattle farms from the Gyeongsang region, including Gyeongbuk (GB) and Gyeongnam (GN) Provinces, between 2018 and 2019 (Figure 3). Data on age, sex, breed, region, and season were documented for each blood sample obtained.

### 4.3. DNA Extraction and PCR

Whole blood samples were used for genomic DNA extraction using the DNeasy Blood and Tissue Kit (Qiagen, Melbourne, Australia) following the given protocol, and the DNA quality and quantity thereof were measured with a NanoDrop™ 2000 spectrophotometer (Thermo Fisher Scientific, Wilmington, DE, USA). To detect hemoprotozoan 18S rRNA, blood samples were first tested for piroplasm infection via PCR using the commercial AccuPower *Babesia* and *Theileria* PCR Kit (Bioneer). Then, positive samples were retested using piroplasm 18S rRNA common primers, BT-F1 (5’-GGTTGATCCTGCCAGTAGT-3’) and BT-R2 (5′-TTGCGACCATACTCCCCCCA-3′) primer set [16] and the MPSP genes, forward (5′-CACGCTATGTTGTCCAAGGAG-3′) and reverse (5′-TGTGAGACTCAATGCGCCTA-3′) primer set, of *Theileria* species via PCR [24]. A negative control, a sample without DNA, was included for each PCR reaction.

### 4.4. DNA Cloning

Purification of the amplified gene fragments were done using the QIAquick Gel Extraction Kit (Qiagen) and then inserted into the pDrive vector (Promega, Madison, WI, USA) according to the given instructions. *Escherichia coli* DH5α competent cells (Thermo Fisher Scientific) were transformed with the resulting constructs. Following bacterial incubation at 37 °C overnight, the Plasmid Miniprep Kit (Qiagen) was used for plasmid purification based on the given instructions.

### 4.5. DNA Sequencing and Phylogenetic Analysis

Recombinant plasmids were sequenced using each 18S rRNA and MPSP gene primers by Macrogen (Seoul, South Korea), and sequences were analyzed using the multiple sequence alignment program *CLUSTAL Omega* (version 1.2.1, http://www.clustal.org/omega/). Results of sequence alignments were modified using *BioEdit* (version 7.2.5, http://www.mbio.ncsu.edu/BioEdit/bioedit.html), and phylogenetic analysis was done with *MEGA* (version 6.0, https://www.megasoftware.net/) using the maximum-likelihood method based on the Kimura 2-parameter distance model. The aligned sequences were evaluated with a similarity matrix. The stability of phylogenetic tree was estimated by bootstrap analysis with 1000 replicates.

### 4.6. Statistical Analysis

Statistical analysis was done using the analytical software package GraphPad Prism version 5.04 (GraphPad Software Inc., La Jolla, CA, USA). The Pearson’s chi-square test was used to analyze tables with more than two variables and the Fisher’s exact test was used to assess 2 × 2 tables. To test which cattle had an increased risk of *T. orientalis* infection within each category, each group was compared with the remaining population enrolled in the study for pairwise comparisons and *p*-values were adjusted using Bonferroni correction. A relative ratio was also calculated. Statistical significance was determined with a *p*-value of ≤0.05 and a 95% CI was determined for all estimates.

## Figures and Tables

**Table 1:** Distribution of bovine *Theileria orientalis* in South Korea in 2018–2019.

Category		No. Tested	No. Positive (%)	RR ^1^	95% CI ^2^	*p*-Value
Sex	Female	327	8 (2.4)	10.0	1.3–79.8	0.0293 *
	Male	410	1 (0.2)	0.1	0.01–0.8	0.0293 *
Age	<2	122	0	0.3	0.02–4.5	0.357
	2–4	413	3 (0.7)	0.4	0.1–1.6	0.1832
	>4	202	6 (3.0)	5.3	1.3–21.0	0.0176 *
Breed	Hanwoo	525	0	0.02	0–0.9	0.0079 *
	Holstein	212	9 (4.2)	46.9	2.7–802.6	0.0079 *
Region	Gyeongbuk	361	0	0.1	0–0.9	0.0451 *
	Gyeongnam	376	9 (2.4)	18.3	1.1–312.3	0.0451 *
Season	Spring	282	0	0.1	0–1.5	0.0886
	Summer	178	9 (5.1)	3.0	1.2–7.5	0.0166 *
	Autumn	277	0	0.1	0–1.5	0.0924
Total		737	9 (1.2)		0.4–2.0	

^1^ RR, relative risk; ^2^ 95% CI, 95% confidence interval. * Statistical significance was assessed by the following Bonferroni-adjusted *p*-values: Sex (<0.025), age (<0.0167), breed (<0.025), region (<0.025), and season (<0.0167).
